# Accuracy of Using Visual Identification of White Sharks to Estimate Residency Patterns

**DOI:** 10.1371/journal.pone.0034753

**Published:** 2012-04-13

**Authors:** David G. Delaney, Ryan Johnson, Marthán N. Bester, Enrico Gennari

**Affiliations:** 1 Oceans Research, Mossel Bay, South Africa; 2 Mammal Research Institute, University of Pretoria, Pretoria, South Africa; 3 South African Institute for Aquatic Biodiversity, Grahamstown, South Africa; Macquarie University, Australia

## Abstract

Determining the residency of an aquatic species is important but challenging and it remains unclear what is the best sampling methodology. Photo-identification has been used extensively to estimate patterns of animals' residency and is arguably the most common approach, but it may not be the most effective approach in marine environments. To examine this, in 2005, we deployed acoustic transmitters on 22 white sharks (*Carcharodon carcharias*) in Mossel Bay, South Africa to quantify the probability of detecting these tagged sharks by photo-identification and different deployment strategies of acoustic telemetry equipment. Using the data collected by the different sampling approaches (detections from an acoustic listening station deployed under a chumming vessel versus those from visual sightings and photo-identification), we quantified the methodologies' probability of detection and determined if the sampling approaches, also including an acoustic telemetry array, produce comparable results for patterns of residency. Photo-identification had the lowest probability of detection and underestimated residency. The underestimation is driven by various factors primarily that acoustic telemetry monitors a large area and this reduces the occurrence of false negatives. Therefore, we propose that researchers need to use acoustic telemetry and also continue to develop new sampling approaches as photo-identification techniques are inadequate to determine residency. Using the methods presented in this paper will allow researchers to further refine sampling approaches that enable them to collect more accurate data that will result in better research and more informed management efforts and policy decisions.

## Introduction

Visual sightings and photo-identification (hereafter this coupled methodology is simply referred to as “photo-ID") is a widely utilized approach [1] as it can produce long-term datasets on a variety of topics such as population size [2]–[4], population demographics of a given population [5]–[7], absolute trends in population numbers [8], and residency [9]–[11]. While photo-ID can collect data on a wide range of topics, the approach is both economically viable and non-invasive [12]. Therefore, photo-ID allows for non-invasive mark-recapture studies, which is critical for threatened species [8] about which we lack sufficient fisheries data [13].

Photo-ID has been used as a monitoring tool on a variety of marine species including marine mammals [14]–[20] and cartilaginous fish such as whale sharks *Rhincodon typus* [21]–[23], nurse sharks *Ginglymostoma cirratum* [24], sand tiger sharks *Carcharias taurus* [25], [26], manta rays *Manta alfredi* [1], [27] and white sharks *Carcharodon carcharias* [2]–[4], [9], [10], [13]. Given the wide use of photo-ID for studying marine animals, photo-ID is arguably one of the most widely used approaches to estimate population size and residency of marine organisms (*i.e.* the amount of time an organism spends in a given area). This is clearly the case for white sharks (1–3, 9, 10, 13). A number of scientists have used photographs of white sharks (*e.g.* their dorsal fins, scars) either during predatory events or at a chumming vessel (*e.g.* cage diving boat, research vessel) to estimate patterns of residency. For example, Klimley and Anderson [9] used visual identification of sharks at an aggregation site during predatory encounters with pinnipeds at the Farallon Islands, California to measure residency. Ferreira and Ferreira [10] used chum to attract sharks to a vessel and then collected tag-visual re-sighting data from Dyer Island and Struis Bay, South Africa. Visual sightings at a commercial cage diving vessel at the Neptune Islands, South Australia were used to describe the potential that the cage diving activity caused conditioning, and suggested that white sharks are temporal visitors to small scale aggregation sites moving through the sites quickly (Robbins R., *pers. comm.*).

Around the world, researchers are beginning to compliment photo-ID with acoustic telemetry. Bruce *et al.* [28] used a VR2 acoustic telemetry array to assess residency of 22 tagged sharks at Dangerous Reef and the North and South Neptune Islands in Australia. Strong *et al.* [29] used both visual identification of individuals visiting a chumming vessel and acoustic tracking for estimating the abundance and residency of a population of white sharks in the lower Spencer Gulf, South Australia. More recently, Laroche *et al.* [30] used a radio acoustic positioning system in conjunction with photo-ID at Seal Island, False Bay in South Africa to assess the impact of chumming and this approach also estimated the presence/absence of white sharks. This research indicated that visual sightings did not accurately reflect visitation patterns of three individuals [30].

Adopting multiple methodologies may allow researchers to evaluate which approach maximizes precision and accuracy for a given objective (*e.g.* estimating residency of a species). In this paper we determined the probability of detection of white sharks from three different sampling approaches and examined if the data produced shows comparable results for residency patterns of white sharks. Then we examined how the sampling intensity affected the accuracy of residency data. Finally, using the results of our experiment, we discuss the advantages and disadvantages of these three approaches and propose how to effectively choose the appropriate method to collect accurate data on white sharks.

## Methods

### Study area

Mossel Bay is a small open bay situated along South Africa's southern coast that is a known aggregation site for white sharks [31]. The bay is approximately 26 km wide and varies in its protection from the open sea. The topography of the bay is dominated by sand bottom, with distinct patches of coastal reef present. Within the inner bay, some 800 m offshore, a small island emerges from the sand bottom and hosts a small population of Cape fur seals (*Arctocephalus pusillus pusillus*) that numbers between 4,500–5,000 individuals (excluding pups of the year) (Kirkman S., *pers. comm.*).

### White shark attraction and photographic identification

White sharks frequently visit chumming boats (*i.e.* either white shark cage diving tourism boat or a research vessel) within Mossel Bay. Therefore on 60 days during our sampling period (*i.e.* 3^rd^ April to 16^th^ November, 2005), which is defined by the first and last time we did the three sampling approaches simultaneously, we traveled to various sites in Mossel Bay and chummed and baited the water with various biological attractants including: the liver of shark by-catch, sardines (*Sardinops sagax*), horse mackerel (*Trachurus trachurus*), several species of tuna and other bony fish (*e.g. Coryphaena hippurus*), fish oil, and/or southern right whale (*Eubalaena australis*) blubber. Once each shark arrived we attempted to photograph each side of its dorsal fin preferably while it was out of the water. These digital images were catalogued in a database using Adobe Light Room, as the basis for determining resighting rates of individuals later in the year, and individuals from past years. Individual sharks were identified by matching notch patterns on the trailing edge of the dorsal fins, scars, and presence of black and white pigment. From this photo-ID catalogue we made sighting histories of white sharks which had been tagged with acoustic RCODE transmitters. Before or during the sampling period 22 sharks were tagged with VEMCO RCODE (VEMCO, Shad Bay, Nova Scotia) passive sensor acoustic transmitters (hereafter simply referred to as “RCODE transmitters"). Each RCODE transmitter was attached at the base of a dorsal fin. Tagged sharks enabled detection and identification using two additional sampling approaches.

### Acoustic telemetry

During our chumming trips where photo-ID was conducted, we deployed a VR2 acoustic listening station from the side of the chumming boat (hereafter referred to as “boat deployed VR2"). Second, a dedicated array of VR2 acoustic listening stations (VEMCO, Shad Bay, Nova Scotia) were deployed since 2001 within the inner section of Mossel Bay (34°07-15′S, 22°06-08′E). During the sampling period between one and six VR2 acoustic listening stations were operating continuously. We collected data from an array of permanently deployed VR2 acoustic listening stations (this arrangement hereafter is referred to as the “array") for the sampling period. The VR2 receivers deployed from the boat and those for the array detected and archived the presence of tagged sharks that moved within a radius of approximately 300–800 m of the chumming vessel depending on water depth and sea surface state. Both the boat deployed VR2 and the array allowed determination of presence of tagged sharks on a daily basis, with individuals considered present in the study site if two or more detection events were archived on any of the receiver(s) on a given day. For deployment of the array, we positioned concrete filled bases (approximately 400–600 kg) on the sea floor, and the listening stations were mounted vertical to these bases. Maintenance and data collection of receivers were carried out every four to nine months. From both the array and the boat deployed VR2, we produced a detection history of tagged sharks, which allows us to estimate the residency of tagged white sharks in Mossel Bay.

### Detection experiment

We examined the sighting rates of tagged sharks for two different sampling approaches (photo-ID and boat deployed VR2). To compare the probability of detection of the two sampling approaches, on 60 days, we simultaneously surveyed the presence of tagged sharks using both the photo-ID and the boat deployed VR2. We recorded the number of tagged sharks detected (*i.e.* sighting rate) by each of the sampling approaches separately. Using these data, we conducted a two-sample t-test to examine if the boat deployed VR2 approach was more effective than photo-ID approach in detecting tagged white sharks.

### Estimating residency experiment

To examine the ability of sampling approaches to estimate residency, we needed to identify the number of sharks with functioning tags on any given day and the period of time that a RCODE transmitter was placed on the shark to last confirmed day that it was retained on the shark and still functioning. The final date of transmission was established from data collected from a number of VR2 arrays deployed in South Africa, including but not limited to: False Bay (Kock A., *pers. comm.*), Gans Bay (Johnson R., unpublished data), and Algoa Bay (Smale M., *pers. comm.*). These areas are within the geographical distribution of South African white sharks, have passive telemetry arrays, and contain Cape fur seal breeding colonies. For the purpose of this study, we further refined the number of days by limiting it to the days counted that fell within the overall sampling period of this study (*i.e.* 3^rd^ April to 16^th^ November, 2005). This number was termed the minimum tag retention time (MTRT), which determined the time period and minimum number of days each shark had a functioning tag during our study. From this, we calculated the total number of sharks with functioning tags on any given day.

To examine the accuracy of sampling approaches in estimating residency of white sharks, we quantified the proportion of days present (PDP) that each shark was confirmed to be in Mossel Bay by each of the three sampling approaches. PDP was calculated as the number of days a shark is detected divided by its MTRT. In addition, for the array data, we also calculated the PDP for each of the tagged sharks based on the data from the complete 228 day array dataset to quantify the increased accuracy based on using the entire sampling effort of the array. This produced four different datasets (60 day photo-ID, 60 day boat deployed VR2, 60 day array, 228 day array) for analysis in a one-way ANOVA with sampling approach as a fixed factor. All statistics in this paper were calculated using MiniTab (Minitab Inc., version 14.1).

## Results

### Detection experiment

**Figure 1:**
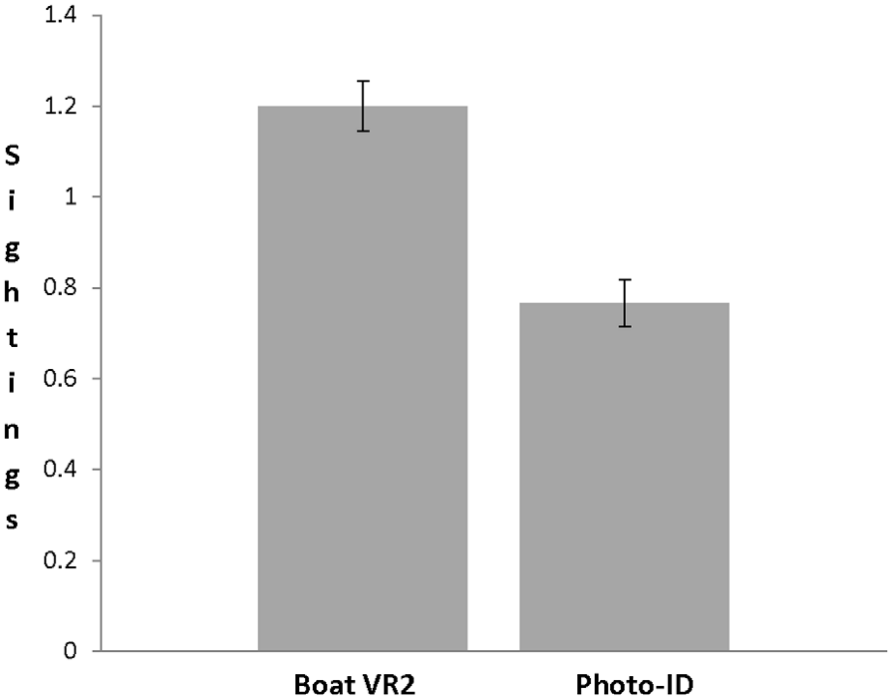
The average number of tagged white sharks detected (“sightings") per day by the two different sampling approaches: photo-ID and a VR2 acoustic receiver deployed from a boat. Values are means ± SE (n = 60 replicates).

The boat deployed VR2 methodology was significantly more effective (*i.e.* higher probability of detection) than photo-ID (P = 0.043, Figure 1). Photo-ID detected 0.767 sharks per day (SD = 0.945, SE = 0.122) compared to 1.20 sharks per day (SD = 1.338, SE = 0.173) for the boat deployed VR2 approach. The number of tagged shark sighted on 60 sampling days ranged from 0–4 and 0–5 for the photo-ID and boat deployed VR2 approaches, respectively.

### Estimating residency

**Figure 2:**
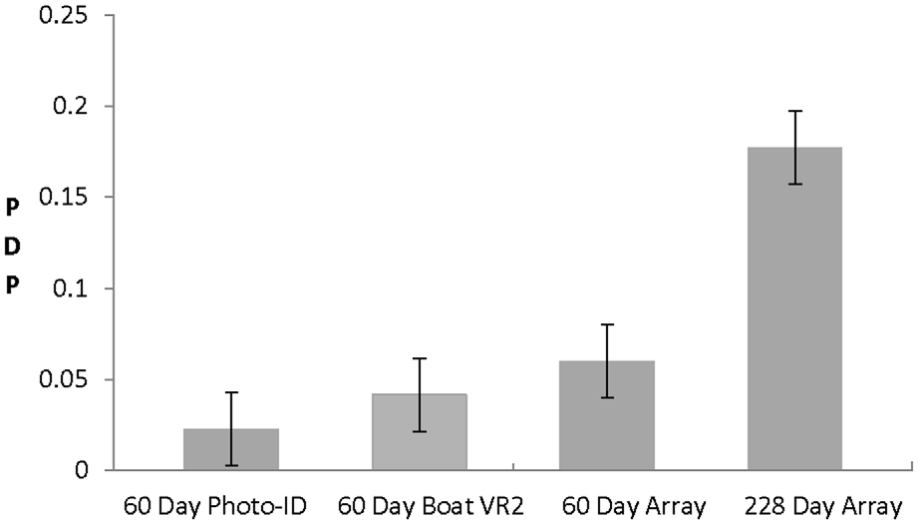
The proportion of days present (“PDP") on average that each shark was confirmed to be in Mossel Bay as determined by each of the three sampling approaches (photo-ID, a VR2 acoustic listening station deployed from a boat, and an array of VR2 acoustic listening stations from 60 days of sampling, and the full array dataset for the entire 228 day study period) during the sampling period (*i.e.* 3^rd^ April to 16^th^ November, 2005). Values are means ± SE (n = 22 replicates).

Eighteen of the 22 sharks with functioning tags during the study were detected by one or more of the sampling approaches in Mossel Bay and the average MTRT for these 22 tagged sharks was 143.4 days during our study period (Table 1).The different sampling approaches gave different estimates of residency for the same tagged white sharks (F_3,84_ = 7.65, P<0.001, R^2^ = 18.7%, Figure 2). Photo-ID estimated the average residency of tagged white sharks as only 2.3% (SD = 5.4%, SE = 1.1%) of the days during our sampling period. The boat deployed VR2 recorded tagged sharks were present on average for 4.2% (SD = 10.5%, SE = 2.2%) of the days during our sampling period. The 60 days of array data indicated that the tagged sharks were present for 6.0% (SD = 10.6%, SE = 2.3%) of the days during the sampling period. While the 228 day array dataset estimated that sharks were present on average on 17.8% (SD = 17.5%, SE = 3.7%) of the days during our sampling period. Due to technical difficulties, the sampling effort of the array varied during the year. The six acoustic listening stations of the array were functioning simultaneously for 23% of the sampling period. The array was reduced to a single unit for 11% of the days during our sampling period but was composed of two or more acoustic listening stations for 89% of the days during our sampling period.

**Table 1:** The minimum tag retention time (MTRT) and estimates of residency by the sampling approaches (photo-ID, a VR2 acoustic listening station deployed from a boat, and an array of VR2 acoustic listening stations from 60 days of sampling, and the full array dataset for the entire 228 day study period) during the sampling period (*i.e.* 3^rd^ April to 16^th^ November, 2005).

Shark#	MTRT	Photo-ID	Boat deployed VR2	60 day array	228 day array
1	228	0.0570	0.0702	0.1009	0.3684
2	103	0.0000	0.0000	0.0000	0.0000
3	223	0.0090	0.0090	0.0269	0.1076
4	228	0.0044	0.0044	0.0044	0.0482
5	228	0.0263	0.0307	0.0482	0.2149
6	169	0.0000	0.0000	0.0000	0.0000
7	88	0.0000	0.0000	0.0227	0.0795
8	81	0.0000	0.0123	0.0370	0.1358
9	70	0.0000	0.0000	0.0000	0.0143
10	124	0.0161	0.0484	0.0645	0.1532
11	160	0.0250	0.0313	0.0625	0.3313
12	158	0.0063	0.0127	0.0316	0.1582
13	4	0.2500	0.5000	0.5000	0.5000
14	189	0.0265	0.0688	0.1164	0.4603
15	163	0.0000	0.0000	0.0000	0.0061
16	163	0.0000	0.0000	0.0184	0.1350
17	106	0.0283	0.0377	0.0566	0.1038
18	163	0.0000	0.0123	0.0613	0.3374
19	75	0.0000	0.0133	0.0267	0.1867
20	160	0.0000	0.0000	0.0000	0.0000
21	117	0.0000	0.0000	0.0000	0.0000
22	154	0.0519	0.0649	0.1429	0.5649

## Discussion

Given human error and limits in the reliability and abilities of technological equipment, no sampling approach has a probability of detection of one. Therefore, researchers need to identify the best sampling approach for a given objective that maximizes the probability of detection. Unfortunately researchers can never be completely confident that they are utilizing the best approach, due to the almost infinite number of sampling approaches. However, our experimental methodology will allow researchers to identify the best approach of those being considered and allow continual refinement of sampling approaches. We have clearly identified that the use of acoustic telemetry is the best sampling approach of the ones we examined in these experiments to estimate residency of white sharks aggregating in specific areas and sampled through photo-ID of individuals attracted to the surface. Therefore, given the higher probability of detection that acoustic telemetry provides, this sampling method collects more accurate data by reducing the probability of false-negatives that occur with the methodology of photo-ID for individuals attracted to the surface.

Photo-ID is probably the most commonly utilized approach for monitoring white sharks and marine mammals. It is has been used to monitor white sharks around the world [1]–[3], [9]–[11], [28], [29]. This technique is particularly applicable to species that are difficult to tag because of their size and intractability [32]. Further, photo-ID is feasible with subjects that do not retain artificial tags for the duration of the evaluation [33], [34]. Also, being a non-invasive sampling technique, it allows for a larger sample size to be obtained (*i.e.* photo-ID of white sharks allows a much larger number of individuals to be monitored than acoustic telemetry does) because it does not require equipment (other than a camera) and prior encounter for tagging is not needed. Therefore, estimating residency by photo-ID is logistically feasible, non-invasive, fairly cost-effective, and is not equipment intensive.

While photo-ID is widely utilized for the aforementioned reasons, our study found data collected by this approach from a chumming vessel could result in a large underestimation of the presence and residency of white sharks. This could occur for the following various reasons: (a) false-negatives, (b) limited time and effort, (c) sharks do not always respond to the chum and/or come to the surface in the presence of a chum trail, (d) sharks do not encounter the chum, or (e) due to a combination of the aforementioned reasons. For the aforementioned reasons, photo-ID is not as effective as using acoustic equipment because acoustic telemetry equipment monitor a large area (as even one receiver can monitor a circle of ∼500 m radius) while photo-ID can only monitor sharks that come to the surface close to the boat. The main drawback of photo-ID used in white sharks is that it relies on sharks being attracted to a chumming vessel and for sharks to come up to the surface long enough for scientists to positively identify individuals. This opens up many biases between individuals such as the difference of motivation to approach the chumming vessel or the behavior potentially affecting capture probability. The types of biases could be very different for terrestrial organisms. Also photo-ID is not as accurate as other approaches. For example, Gubili *et al.* [35] documented that data from photo-ID contains more errors than another sampling approach (genetic sampling) and Laroche *et al.* [30] and Weng *et al.* [36] also cast doubt on the reliability of photo-ID to record accurate data.

The reliability issues in residency data collected by photo-ID can be avoided by utilizing other sampling methods such as acoustic telemetry. Acoustic telemetry can collect data that is useful for various purposes, including documenting the ecology, movement, habitat use, physiology and residency of white sharks and other species. In our experiments we found that acoustic telemetry equipment is more effective than photo-ID in estimating the residency of white sharks in Mossel Bay, South Africa. Research elsewhere and similar experiments with other species (*e.g.* marine mammals and other species of sharks) would need to be conducted to confirm the generalization of this conclusion but studies on white sharks off the coast of North America and South Africa support the results of our study [30], [36].

Acoustic telemetry is not only a more effective approach but it provides more complete datasets and is less labor-intensive. When functioning properly, the acoustic receivers record continuously 24 hours a day, 7 days a week, including days with rough seas, which are periods that are not safe for researchers to conduct surveys at sea and this allows for more accurate estimates of residency. For example, the dataset for 60 days of array data that documented residency of the tagged white sharks in this study was 2.6 times higher than the measure estimated from the dataset collected from photo-ID monitoring. The 228 days of array data yielded a residency measure for the same white sharks that was 7.8 times higher than the value estimated from photo-ID. This illustrates that data collected from acoustic telemetry equipment provides more accurate estimate of residency than can be estimated from photo-ID but this is due to the ability of acoustic listening stations to monitor for more time and also over a larger area than photo-ID sampling approach can.

On the other hand, there are also limitations to using acoustic receivers and telemetry arrays. In our study, transmitters were deployed externally to the base of sharks' dorsal fins. This approach is less invasive but can lead to tags being damaged, shed or otherwise removed, which reduces sample size and prematurely stops data collection and if occurs frequently can even cause the data collected to be treated as unreliable or only used with caution (1, 37). This can be avoided by using internal tags [1], [32], [37]–[40], which prevent biofouling and tag shedding but is more invasive. Acoustic tags and listening stations are expensive. The latter sometimes are deployed in conditions that can be damaging to the devices (*e.g.* biofouling organisms that settle on the equipment, corrosive salt water, and storms). Malfunctioning devices can greatly delay or impair data collection. Also equipment repairs can be expensive and time consuming. Increasing the number of acoustic listening stations can increase the probability of detection. It also increases the cost of the research and the likelihood that acoustic listening stations will fail. This array was only complete and functioning for less than a quarter of our study period and given these problems, our array of acoustic telemetry equipment, on average, did not provide data on the sharks over 80% of the time. This is probably also due to the large home ranges and transoceanic migratory behaviors of white sharks [11].

Given the global distribution, large-scale, and even transoceanic migrations of white sharks, research teams need to increase the sampling intensity and geographical coverage of acoustic telemetry arrays to accurately estimate residency and document large-scale movement patterns. To achieve this will require additional funding, equipment, collaboration between biologists and data sharing of scientists around the world that use acoustic telemetry equipment to increase their ability to detect and properly study species, such as, white sharks. We recommend that future research examines how to optimally allocate limited equipment in a given study area (*e.g.* a bay, an ocean, the planet) [41]. While acoustic equipment can fail and our acoustic telemetry array was highly variable in the number of functioning units, it still provided the most complete and accurate measure of residency. Also given the improvement in technology of receivers and acoustic listening stations, this approach is becoming more effective and reliable. Given the results of this study, this technology will hopefully become the most common way to estimate residency of marine species.
